# Echocardiography-based AI for detection and quantification of atrial septal defect

**DOI:** 10.3389/fcvm.2023.985657

**Published:** 2023-03-10

**Authors:** Xixiang Lin, Feifei Yang, Yixin Chen, Xu Chen, Wenjun Wang, Wenxiu Li, Qiushuang Wang, Liwei Zhang, Xin Li, Yujiao Deng, Haitao Pu, Xiaotian Chen, Xiao Wang, Dong Luo, Peifang Zhang, Daniel Burkhoff, Kunlun He

**Affiliations:** ^1^ Medical Big Data Center, Chinese PLA General Hospital, Beijing, China; ^2^ Medical School of Chinese PLA, Beijing, China; ^3^ BioMind Technology, Beijing, China; ^4^ Department of Pediatric Cardiac Center, Beijing Anzhen Hospital, Capital Medical University, Beijing, China; ^5^ Department of Cardiology, The Fourth Medical Center of Chinese PLA General Hospital, Beijing, China; ^6^ Department of Ultrasonography, The Sixth Medical Center of Chinese PLA General Hospital, Beijing, China; ^7^ Cardiovascular Research Foundation, New York, NY, United States

**Keywords:** artificial intelligence, deep learning, echocardiography, atrial septal defects, congenital heart disease

## Abstract

**Objectives:**

We developed and tested a deep learning (DL) framework applicable to color Doppler echocardiography for automatic detection and quantification of atrial septal defects (ASDs).

**Background:**

Color Doppler echocardiography is the most commonly used non-invasive imaging tool for detection of ASDs. While prior studies have used DL to detect the presence of ASDs from standard 2D echocardiographic views, no study has yet reported automatic interpretation of color Doppler videos for detection and quantification of ASD.

**Methods:**

A total of 821 examinations from two tertiary care hospitals were collected as the training and external testing dataset. We developed DL models to automatically process color Doppler echocardiograms, including view selection, ASD detection and identification of the endpoints of the atrial septum and of the defect to quantify the size of defect and the residual rim.

**Results:**

The view selection model achieved an average accuracy of 99% in identifying four standard views required for evaluating ASD. In the external testing dataset, the ASD detection model achieved an area under the curve (AUC) of 0.92 with 88% sensitivity and 89% specificity. The final model automatically measured the size of defect and residual rim, with the mean biases of 1.9 mm and 2.2 mm, respectively.

**Conclusion:**

We demonstrated the feasibility of using a deep learning model for automated detection and quantification of ASD from color Doppler echocardiography. This model has the potential to improve the accuracy and efficiency of using color Doppler in clinical practice for screening and quantification of ASDs, that are required for clinical decision making.

## Introduction

It is estimated that in 2017, nearly 1.8 cases per 100 live births are diagnosed with congenital heart disease (CHD) worldwide (1). Atrial septal defect (ASD) is the second most common type of CHD, accounting for approximately 6%–10% of cases (2). Most patients with ASD are asymptomatic and may be identified as an incidental finding during routine echocardiographic examinations. Early detection of appropriately sized defects known to lead to problems later in life can prompt timely intervention and improve cardiovascular outcomes, avoiding substantial disability and mortality (3, 4).

Transthoracic echocardiography (TTE) with Doppler flow imaging is currently the most widely used noninvasive tool for detecting the presence of an ASD, especially in children (5). TTE cannot only be used to detect and quantify the size and shape of the septal defect, but can also be used to measure the degree and direction of shunting, changes of the size and function of the cardiac chambers and detect abnormal pressures and flows through the pulmonary circulation (6). However, accurate detection and quantification of ASD features relies on experienced, highly trained physicians which are in short supply, especially in rural areas (7). Furthermore, the low prevalence of disease and variability of image quality, number of acquired views and interpretation of TTE images causes low sensitivity and specificity of ASD detection (4), all of which hinder referral for treatment. Therefore, an effective solution for efficient, accurate and objective detection and grading of ASDs is critically needed.

**Figure 1:**
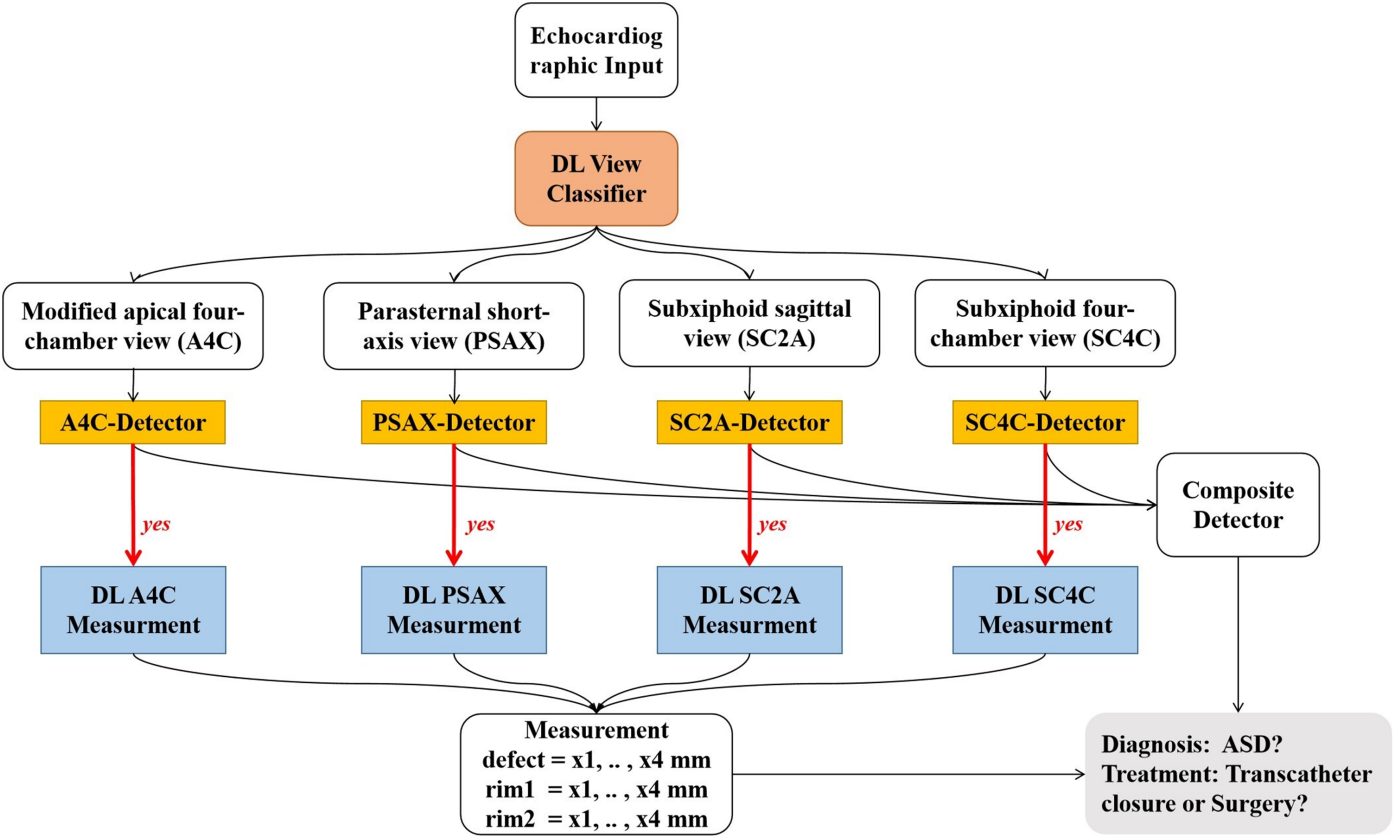
Work flow of the ensemble model. Step 1: raw echocardiographic videos are separated for classification of views (red box). Step 2: disease detection models use different views to detect the presence of ASD (orange rectangles). Step 3: if ASD is present (denoted by “yes”), metrics associated with severity of ASD are assessed (blue rectangles). DL, deep learning; ASD, atrial septal defect.

Deep learning (DL) models have been applied for automated detection and assessment of cardiovascular diseases based on echocardiographic images and videos. Such models can complete a variety of tasks such as, image quality assessment, view classification, boundary segmentation, disease diagnosis and automatic quantification (8–12). However, there is no prior study investigating the effectiveness of a DL model for detecting ASD based on color Doppler images. Accordingly, we developed and validated a DL model for automated detection and quantification of ASDs (Figure 1).

## Methods

### Study population

This study involved algorithm development and initial testing based on a retrospective data set, and final testing from a prospective, real-world data set of consecutively acquired echocardiographic studies. 396 TTE examinations obtained between July 2020 and April 2021 from Anzhen hospital served as our training dataset. A total of 425 consecutively obtained examinations between May 2020 and Dec 2020 from the Chinese PLA General Hospital were collected as the external testing set, which including 48 ASD cases and 377 cases without ASD. The age of all cases in both training and testing datasets was less than 18. ASD diagnostic criteria were based on the 2015 ASE guideline (6), as detailed below. The ground truth for the presences of an ASD was based on the diagnosis present in the electronic medical record and echocardiographic clinical report which were provided by experienced echocardiography readers and reviewed by cardiologists who authorized the final reports. Other hemodynamically significant cardiac lesions (such as tetralogy of fallot and valvular heart disease) were excluded.

### Echocardiography

Each echocardiographic study was acquired through standard methods. Four standard views were suggested by ASE guideline for detection and quantification of ASDs (6): (1) the modified apical four-chamber view (A4C); (2) the parasternal short-axis view (PSAX); (3) the subxiphoid sagittal view (SC2A); and (4) the subxiphoid four-chamber view (SC4C). These images were acquired from a diverse array of echocardiography machine manufacturers and models including Phillips iE-elite and 7C with transducer S5-1 and X5-1 (Phillips, Andover, MA, USA), Vivid E95 (General Electric, Fairfield, CT, USA), Mindray M9cv with transducer SP5-1s (Mindray, Shenzhen, Guangdong, China), Siemens SC2000 with transducer 4V1c (Siemens, Munich, Germany). All images were downloaded and stored with a standard Digital Imaging and Communication in Medicine (DICOM) format according to the instructions from each manufacturer.

### View selection

We labeled 3,404 images to develop a method to classify 29 standard views, and then selected the 4 views required for detection and quantification of ASD detailed above. View selection was performed using a Xception Net neural network model according to methods that were similar to those described previously (8, 10, 13).

### Segmentation

**Figure 2:**
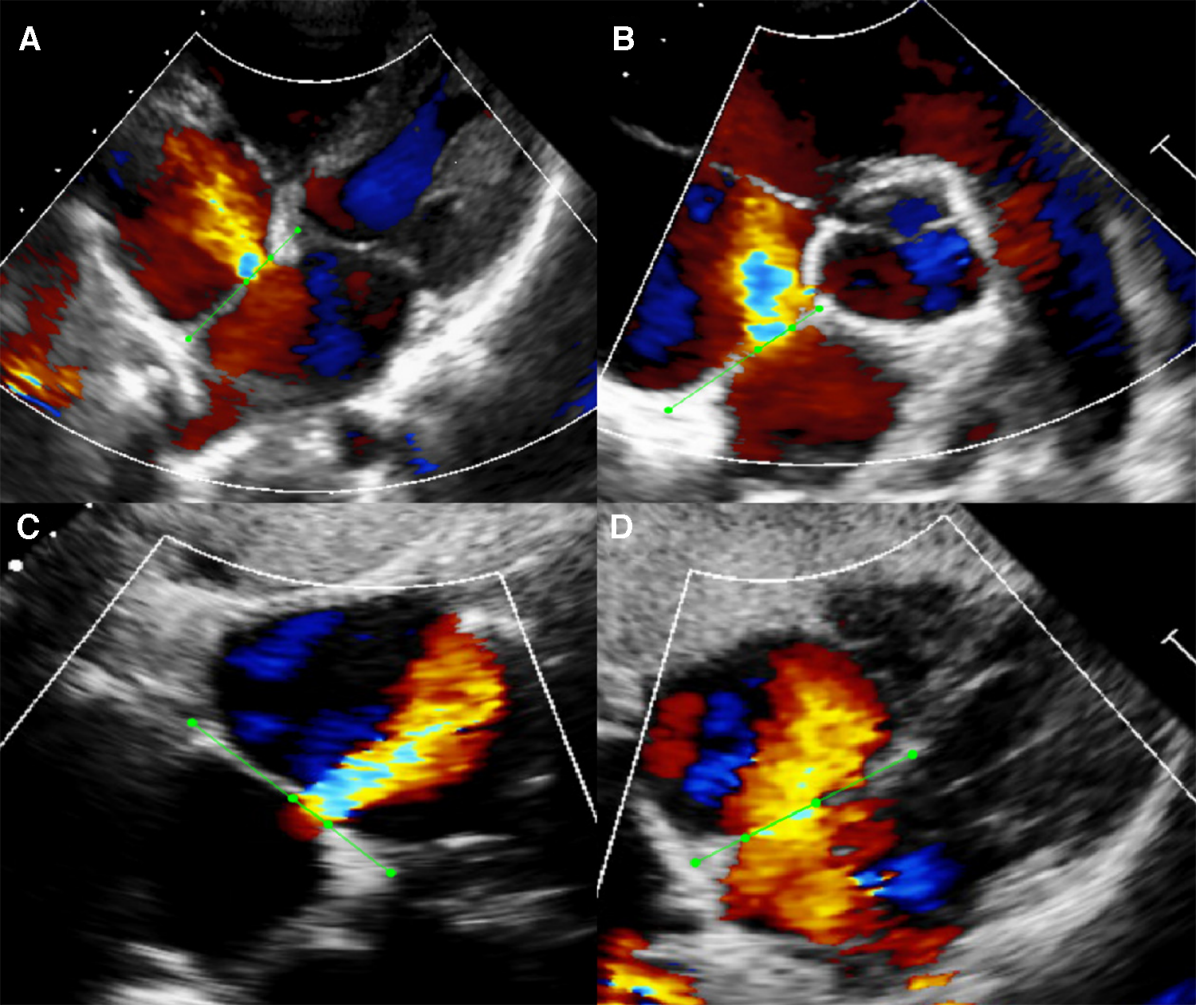
Example of manual segmentation. Green dots were manually labeled as the endpoints of atrial septum and defect with the open-source program LabelMe. (**A**) Modified apical four-chamber view (A4C). (**B**) Parasternal short-axis view (PSAX). (**C**) Subxiphoid sagittal view (SC2A). (**D**) Subxiphoid four-chamber view (SC4C).

We selected 792 videos inclusive of the four standard color Doppler views required for segmentation from among the ASD cases. However, not every case had all four standard views, restricted by the limitation of retrospective data and the improper body position during examination. The atrial septum and margins of the defects were annotated with the LabelMe (Figure 2). In the modified apical four-chamber and subxiphoid four-chamber views, we labelled the atrial septum from atrioventricular valve to the roof of the atria (boundaries indicated by the dots in Figure 2). In the parasternal short-axis view, we labelled the atrial septum from aortic adventitia to the roof of the atria. In the subxiphoid sagittal view, we labelled the atrial septum from the bottom to the roof of the atria. We labelled the defect based on the width of the shunt jet detected on color Doppler flow images and the anechoic area of atrial septum in each view.

### Detection of atrial septal defect

For the ASD detection task, videos were labelled as either ASD or normal based on the electronic medical record and echocardiographic clinical report. Each frame was resized to 240 × 320 pixels from 600 × 800 DICOM-formatted images. The pixel value was normalized to between 0 and 1. No clipping or interpolation operations were performed on frame numbers; therefore, the number of frames used for the analysis differed from video to video. To effectively increase the number of videos for training, we employed affine transformations including RandomShift (10%), RandomScale (10%) and RandomRotation (20°). The batch size was set to 1 because of the difference of frame number. Finally, we adopted the Adam optimizer with a weight decay of 1e-5. The learning rate was set to 3e-5. All models were trained on an Nvidia Tesla P100 GPU.

**Figure 3:**
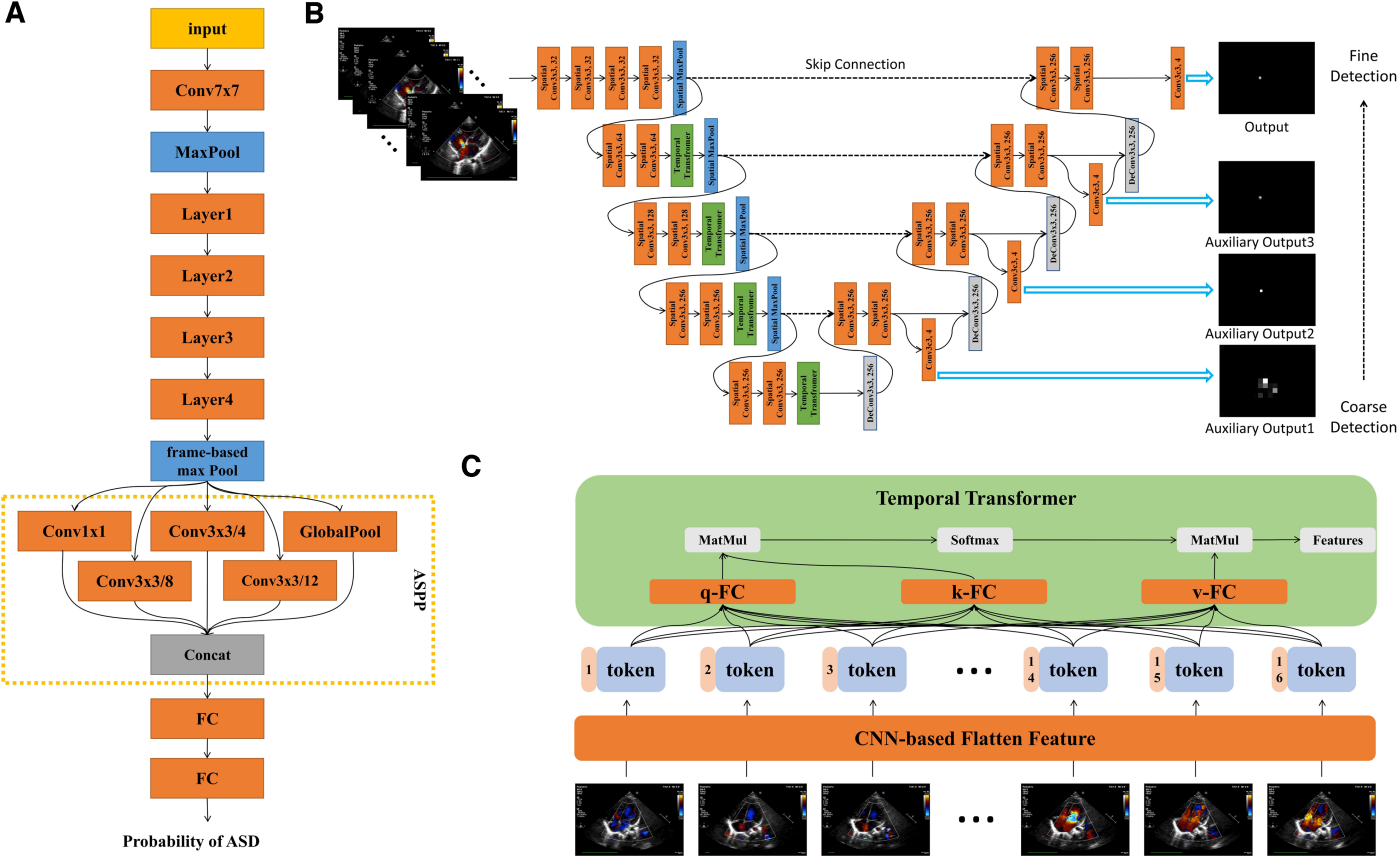
Detailed description of model architecture. (**A**) ASD detection neural network architecture, based on Resnet-18, combined with ASPP to increase visual field. The output of the model is the probability of ASD between 0 and 1. (**B**) ASD quantification neural network architecture, combining spatial convolution and temporal transformer, had three auxiliary outputs and one output from coarse to fine. Each auxiliary output is added to the feature map as an additional feature. (**C**) Structure diagram of temproal transformer. Each frame is encoded into a feature token by CNN. Self-attention could realize the long-distance dependence between frames. *q*, query; *k*, key; *v*, value.

The ASD detection network architecture was shown in Figure 3A. The model was based on the ResNet architecture with modifications (14). First, we used a frame-based max pool to fuse blood flow information in each frame. Second, we used Atrous Spatial Pyramid Pooling (ASPP) to increase the visual field of the convolution feature extractor. ASPP consisted of a global average pool layer and four convolution layers with dilation coefficients of 1, 4, 8 and 12 respectively. Third, we used GroupNorm to replace BatchNorm since the batch size was 1. The loss function was binary cross-entropy. Finally, the model could provide the ASD probability of each frame in the video. Therefore, the frame with the highest probability would be selected as the keyframe of model diagnosis. We have made our code available at GitHub (15).

### Quantification of atrial septal defect

For the quantification tasks, each frame was labelled with 4 points, two of which were the edges of the ASD, and the other two were the ends of the septum. Each frame was resized to 240 × 320 pixels. The pixel value was normalized to between 0 and 1. For the training stage, we randomly clipped 16 consecutive frames as an input from the video. Because of the large number of training epochs, we believed that the model had fully learned the entire information for each training sample. For validation and testing stage, each video was clipped into multiple segments. According to the majority voting principle, we made the prediction from those of segments. Each prediction took into account all the video information and did not receive the impact of randomness. We adopted the same affine transformation described above. In this case, the batch size was set to 2. We adopted Adam optimizer with a weight decay of 1e-5. The learning rate was set to 3e-5. All models were trained on an Nvidia Tesla P100 GPU.

The quantification network architecture was shown in Figure 3B. The networks architecture adopted UNet-style design (16). There were two dilemmas in using deep learning to fulfil the ASD quantification task.

Firstly, measuring the length of ASD was defined as a segmentation task, so we also adopted the structure of 3D-UNET (16). However, the performance was far less than expected. Segmenting the area of atrial septal defect was not a routine segmentation task. The common segmentation task assumes a segmentation boundary in the image, but the atrial septal defect was a disappearing region. Experienced doctors need to annotate the region based on sequent images. Therefore, we defined the task of quantifying ASD as key point detection since each key point exists in the image. We continued 3D-Unet-style and applied it to the task of key point detection.

The second point was how to make the neural network perform similarly to the senior physician. We have made two improvements: we have added different scales of auxiliary loss so that the model was from coarse to fine for key point identification; we found that the temporal convolution was unsuitable for the ASD key point detection task in echocardiography and therefore replaced it with a temporal transformer to overcome the long-range dependence dilemma of the frame dimension. The dependency dilemma can be described as follows: the key point detection of unclear video frames depends on the information obtained from the previous and next video frames. Such unclear video frames were present in human pose detection (17), for example, in the form of occlusion or overlap. However, this occlusion or overlap exists for a short period of time, usually no more than 3 consecutive frames. In echocardiography, most of the frames were not clear enough. Therefore, an experienced sonographer will prioritize the key points in clear video frames and then identify key points in other frames that were not clear enough. Convolution was a natural local attention mechanism and was not global, so using convolution to extract features will suffer long-range forgetting dilemma. As a simple example, suppose we use a 3 × 3 × 3 convolution kernel for 3D convolution and we want the features of frame *i* to fuse the features of frame *j*. If |i−j|≤1, then the features of *i* and *j* were ready for fusion in the 1st convolutional layer. If |i−j|≤3, then the features of *i* and *j* need to go to the 3rd convolutional layer before they can be fused with each other. If the distance between frame *i* and frame *j* was long, the long-range forgetting dilemma will occur. In order to allow the features of clear video frames to be efficiently propagated throughout the whole video, we use the temporal transformer module. The structure of temporal transformer was shown in Figure 3C. Firstly, CNN-based extractor extracted the feature from each frame. In each temporal transformer module, the feature was transferred to three parts, query (*q*), key (*k*) and value (*v*). We calculated the correlation between *q* and *k*, which was called self-attention. We had 16 frames (tokens) so that the correlation map was 16 × 16. This map represented the correlation between any two frames. Finally, we used the correlation map processed by softmax as the weight, and sum the value. For the whole model, we only downsampled in the spatial dimension, and did not downsample in the temporal dimension so that for each temporal transformer block, the token number was always 16.

In addition, we have made the following adjustments: considering the cost of computation and time, we used the 2D spatial convolution and temporal transformer to replace the 3D convolution. Second, we used GroupNorm to replace BatchNorm since the batch size was small. Video $x\in R^{W\times H\times F}$ (*W* means width, *H* means height and *F* means frame) goes through convolutional layers for feature extraction in *W* and *H* spatial dimensions. Then the *W* and *H* dimensions were merged into token *T* so feature map can be writen as $f\in R^{W{}^{\prime }\times H{}^{\prime }\times F}$ or $f\in R^{T\times F}$. The token of each frame was spliced with the corresponding position code, and then can be used as the input of the temporal transformer. The self-attention mechanism follows the design of ViT (18). The model can be divided into 4 stages, each containing a spatial downsampling layer, spatial convolution layers and a temporal transformer module.

The model provided an index of the “confidence” with which the septal length was estimated. Confidence was calculated as the percent of “stable frames” contained in the entire video. A frame was designated as “stable” if the absolute difference of the AI-predicted septal length from that of the prior frame divided by the average length of the 2 frames was less than 0.5. The model only calculated defect size and septal length based on the stable frames. Specifically, septal length was calculated as the average value of the lengths on all stable frames. ASD defect size was calculated the largest value among all stable frames. Accuracy of measurements of atrial septal lengths and defect sizes were compared to those made by expert echocardiographers using Bland & Altman analysis.

### Statistical analysis

Analyses were performed using algorithms written in Python 3.6 from the libraries of Numpy, Pandas, and Scikit-learn. Continuous variables were expressed as mean ± standard deviation, median and interquartile range, or counts and percentage, as appropriate. Comparisons of reports and machine algorithm performances were performed using one-way analysis of variance (ANOVA), followed by the least significant difference (LSD) *t*-test. Results were regarded as statistically significant when *P* < 0.05. The models were assessed according to the area under the receiver operating characteristic (ROC) curves which plotted sensitivity vs. 1-specificity derived from the model's prediction confidence score. All calculations were performed by using IBM SPSS version 23.0.

## Results

### Characteristics of study population

A total of 821 patients with transthoracic echocardiographic examinations were included. The clinical and echocardiographic characteristics of included cases were summarized in Table 1. In the training dataset, patients with ASD had a median age of 3 years (IQR: 1, 10), 34.3% were male, and EF had a mean value of 70.0 ± 5.0. In the external testing dataset, patients with ASD had a median age of 1 years (IQR: 0, 9), 52.0% were male, and ejection fraction (EF) had a mean value of 64.7 ± 5.3.

**Table 1:** Baseline characteristics of the training and testing dataset.

	Training dataset		Testing dataset	
	ASD	Control	ASD	Control
*N*	198	198	48	377
Age (years)	3 (1,10)	3 (1,6)	1 (0,9)	5 (1,13)
Male patients (%)	68 (34.3)	105 (53.0)	25 (52.0)	238 (63.1)
Height (cm)	112.6 ± 34.8	107.4 ± 28.3	88.6 ± 35.5	125.2 ± 35.2
Weight (kg)	26.9 ± 21.3	22.2 ± 14.7	16.5 ± 20.5	30.0 ± 22.5
Echo parameters				
LV EF (%)	70.0 ± 5.0	70.0 ± 5.3	64.7 ± 5.3	65.6 ± 3.5
LV EDD (mm)	30.8 ± 7.4	34.6 ± 6.8	23.4 ± 9.8	33.4 ± 8.4
LV ESD (mm)	19.1 ± 6.5	21.0 ± 4.6	14.5 ± 6.9	20.7 ± 5.8
LA AD (mm)	21.8 ± 7.3	21.0 ± 5.0	16.4 ± 7.2	22.1 ± 5.6
E/A	1.7 ± 1.5	1.7 ± 0.5	1.6 ± 0.5	1.7 ± 0.5

ASD, atrial septal defect; LVEF, left ventricular ejection fraction; LV EDD, left ventricular end-diastolic dimension; LV ESD, left ventricular end-systolic dimension; LA AD, left atrial anteroposterior dimension.

### Model for view selection

As summarized in Supplementary Figure S1, the deep-learning model identified four standard color Doppler views with an average accuracy of 0.99, including apical four-chamber view (0.97), parasternal short-axis view (0.99), subxiphoid frontal view (0.99) and subxiphoid sagittal view (1.0).

### Model for detection of atrial septal defect

**Figure 4:**
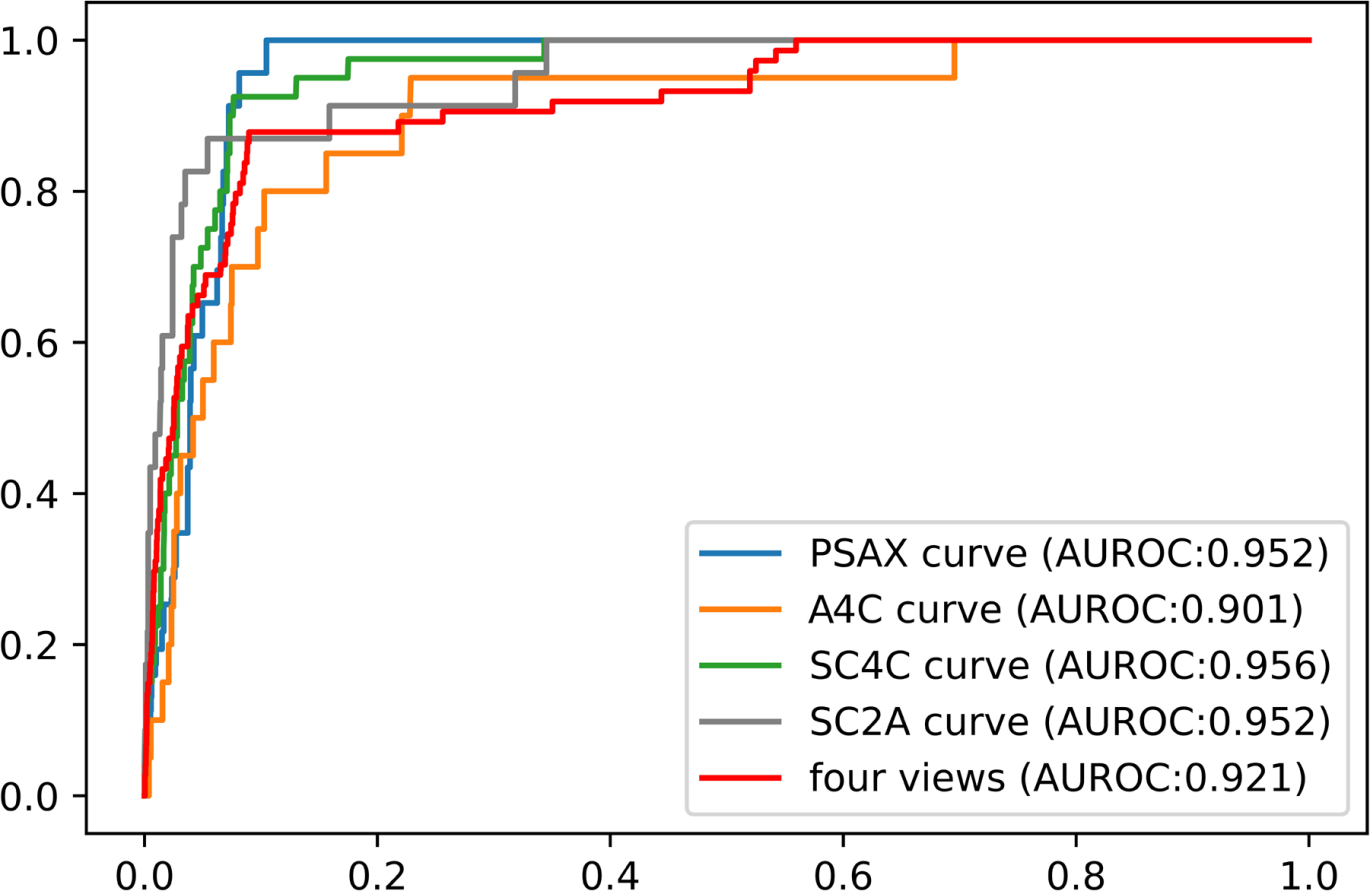
The performance of ASD detection model in the external dataset. The performance of composite classifier model (red curve) had an AUROC of 0.92. Abbreviations as in Figure 1.

For each echo-Doppler video, the ASD detection model provided a probability level for the presence of an ASD; the frame with the highest probability was tagged as the keyframe of the video (examples shown in Supplementary Figure S2). The ROCs for the detection of an ASD in each of the 4 views for the external validation dataset were shown in Figure 4. The AUROC for ASD detection ranged from 0.901 to 0.956 for the individual views. The final diagnosis was made by the composite classifier model, which had an AUROC = 0.92. Youden's Index was used to evaluate model performance, which yielded sensitivities of 87.8% and specificities of 89.4% (Figure 4 and Table 2).

**Table 2:** Model performance for ASD detection in different views.

	AUC	Sensitivity	Specificity
Composite	0.921	87.8%	89.4%
A4C	0.901	85.0%	84.4%
PSAX	0.952	95.7%	91.9%
SC2A	0.952	91.3%	83.8%
SC4C	0.956	92.5%	92.3%

Abbreviations as in Figure 1.

### Model for quantification of atrial septal defect

**Figure 5:**
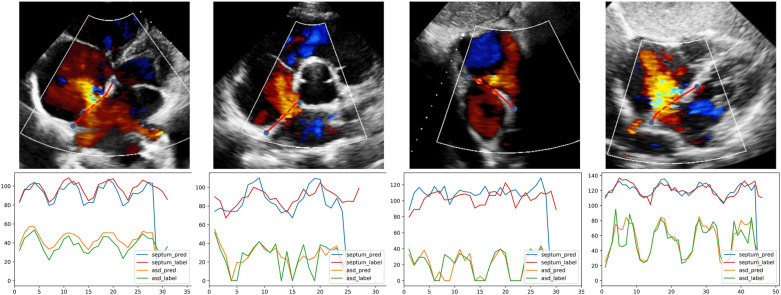
The output of quantification model in different views. The first row, one example test image of patients with ASD is shown respectively in A4C, PSAX, SC2A and SC4C view; orange and blue dots are the endpoints of defect and septum predicted by DL model. The second row, corresponding curves showing the variation of defect and septum in the video. Abbreviations as in Figure 1.

Examples of segmentation model outputs were shown in the still image of Figure 5. As shown, the blue dots show where the DL model identified the ends of the atrial septum, while the orange dots show the model-identified edges of the ASD. Examples of frame-by-frame segmentation throughout entire videos, along with the model-derived measurements of the defect size and rim lengths compared to those measured by the expert physicians were shown in the videos provided in with online supplement material. These videos show results obtained from different echocardiographic views and different image qualities.

**Figure 6:**
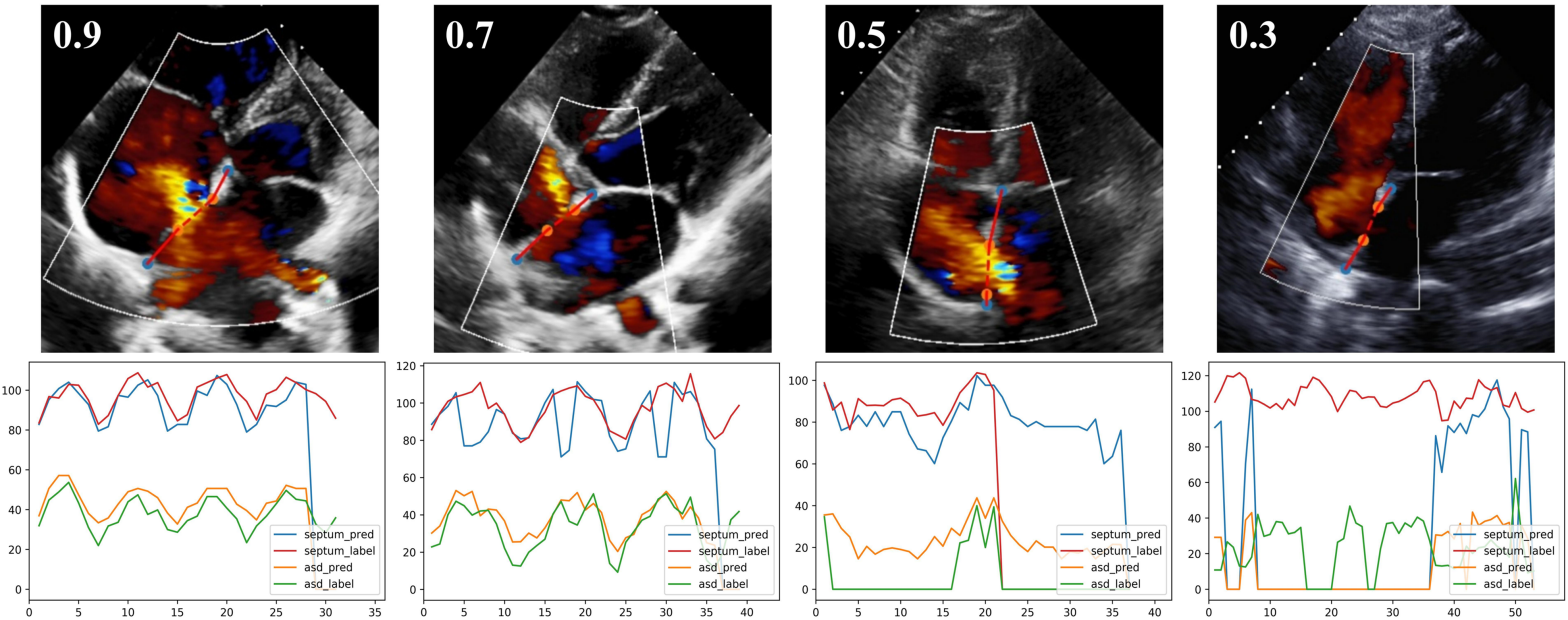
The output of quantification model with different confidence levels. The first row, one example test image of A4C view is shown respectively with the confidence of 0.9, 0.7, 0.5 and 0.3. The second row, corresponding curves showing the variation of defect and septum in the video.

As detailed in Methods, the model provided an index of the “confidence” with which the septal length was estimated. Examples of results with different confidence levels were shown in Figure 6. The quantification model had greater performance in videos with higher confidence values; the relationships between the absolute difference between AI- and expert-determined septal length and ASD lengths as a function of the confidence values were shown in Supplementary Figure S3.

**Figure 7:**
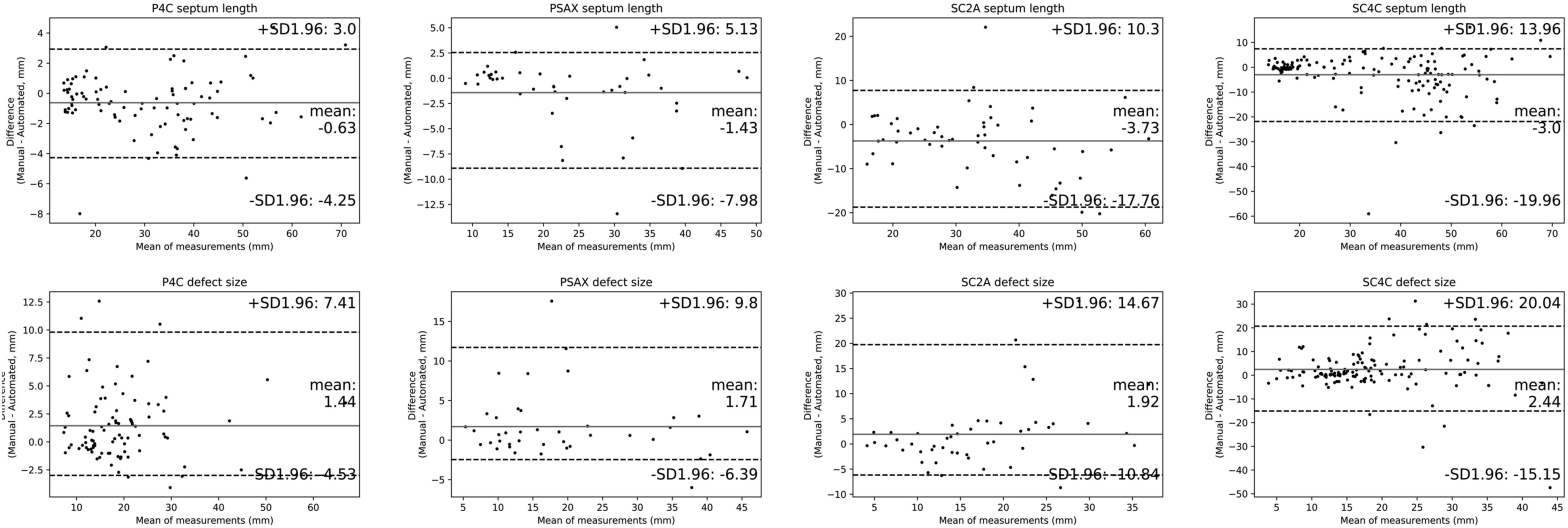
Comparisons of quantitative metrics derived from the deep learning (DL) algorithm and physician based on bland and altman analysis. Bland-Altman plots compare automated and manual measurements for septum length and defect size in A4C, PSAX, SC2A and SC4C view. Abbreviations as in Figure 1.

Results of the Bland & Altman analysis comparing values provided by the AI algorithm and experts' measurements were summarized in Figure 7. The mean bias for the measurement of defect size and septum length were 1.9 and 2.2 mm. We also recruited three experts to measure defect size and septum length in the test dataset. As shown in Supplementary Figure S4, the mean biases of defect size were respectively 1.5 mm, 2.3 mm, 0.3 mm, and the mean biases of septum length were respectively 0.8 mm, 2.1 mm, 1.2 mm. Despite the fact that inter-expert variability was lower than the AI model bias, the difference was insignificant. Therefore, we believed that the bias of algorithm is comparable to that encountered in current clinical practice.

Applying these automatic measurements to the indications and contraindications detailed in the 2015 ASE guidelines, we used the model to predict whether a given patient should be referred for transcatheter intervention (6). The results of the prediction were compared with the recommendations provided by an expert physician, who applied his own manual measurements to the guideline recommendations. The accuracy of model to predict the expert's conclusion was 85.4% (Supplementary Table S1).

## Discussion

Echocardiography is the primary method for confirming the diagnosis of an ASD, for defining its anatomic and physiological characteristics and for deciding upon the need for and approach to treatment. However, accurate interpretation of echocardiograms for each of these purposes is in many respects subjective and time-consuming, requiring highly skilled clinicians which are not readily available in all hospitals. With the advantages of objectivity, efficiency, accuracy and consistency, deep learning (DL) models have been shown to be helpful in interpreting medical images in many fields of medicine (19–21), including echocardiography (8–12). However, ours is the first study to employed DL model for accurate detection and quantification of ASD through automated interpretation of color Doppler videos.

As in most DL models applied to echocardiography, the first step in our pipeline was echocardiographic view classification (22–24). However, newly introduced in our study is a classification model that includes color Doppler views. This model automatically selected the guideline-recommended echocardiographic views required for the detection and quantification of ASD with a high degree of accuracy.

The next step was implementation of a DL model to detect the presence of an ASD based on interpretation of the color Doppler views. This model also proved to be accurate, with high levels of sensitivity and specificity for disease detection. Similar degrees of detection accuracy were reproduced in all four echo-Doppler views examined and the AUC of the composite classifier model reached to 0.92. In addition, to address the “black box” problem and improve the interpretability, our model also automatically identified the key frame which can be provided to the clinician as a reference for final diagnosis and manual verification. Accordingly, the model has the potential to be used as a screening tool to aid doctors in identifying patients with an ASD, particularly in geographies where access to expert clinicians is limited.

Following view selection and disease detection, the final step was automated quantification of ASD size and the length of the residual rim; these are critical for determining the need for, and choice of treatment: transcatheter intervention or cardiothoracic surgery. To make these measurements, the quantification model automatically located the endpoints of the atrial septum and of the defect. In order to ensure the stability and reliability of automated quantification, the model generated an index of “confidence” with which the septal length was estimated. Naturally, the quantification model had greater performance in videos with higher confidence values. The performance of the algorithm was assessed by the bias of measurement of defect size and septum length, which provided a quantitative index of the degree of concordance between the DL model and expert physicians. Values of bias achieved by the model were low. Because the model explicitly detected and displayed the location of endpoints of the septum and defect, physicians can readily verify the accuracy of the DL algorithm on a case-by-case basis. All these features are illustrated in the videos provided on the online supplemental material.

Finally, we note that whereas the metrics of defect and rim size are helpful for deciding between use of a transcatheter or surgical intervention, such decisions are not made based on these metrics alone. According to society guidelines (6) such decisions should be made based on additional metrics and other imaging approaches, such as transesophageal echocardiography and three-dimensional imaging. While this tool has potential utility in areas where access to expert physicians is limited, the method was trained and validated on images acquired by experts. So its translation to resource-limited environments might require additional adaptations to give real-time feedback on image quality if datasets are acquired by individuals with limited specialization in echocardiography of congenital defects.

### Related work

Recent studies have shown remarkable performance of deep learning models in diagnosing ASDs (25–28). Wang et al. proposed an end-to-end framework which automatically analyzed multi-view echocardiograms and selected keyframes for disease diagnosis. As a result, the framework differentiated ASD, VSD and normal cases with an accuracy of 92.1% (25). Rima et al. used fetal screening ultrasound to train a DL model for these tasks, including view selection, segmentation and complex congenital heart disease detection. In the test of 4,108 fetal sonograms, the model achieved an AUC of 0.99 in distinguishing normal from abnormal hearts, which was comparable to expert clinicians' performance (26). Zhao et al. developed a variant of U-Net architecture to segment the structure of the atrial septum in magnetic resonance images of pre- and post-occlusion ASD patients, with mean Dice index of 0.81 (27). Mori et al. proposed a DL model that used electrocardiograms (ECGs) to detect the presence of ASDs. This model outperformed 12 pediatric cardiologists in diagnosing ASD from ECG interpretations, with an accuracy of 0.89 (28). However, ours is the first study to detect the presence of an ASD based on multiple color Doppler views and to automatically identify the margins of the atrial septum and the margins of the ASD in order to provide quantitative measurements of ASD size and rim size. These represent significant advances since quantification of these anatomic features of an ASD are critical for determining treatment. Specifically, according to the 2015 ASE guideline (6), echocardiography provides important information for deciding on whether or not to treat an ASD and whether the defect is most suitably treated by transcatheter or surgical techniques. In this regard, studies have shown that ASD diameter measured directly at surgery is most accurately estimated by color flow Doppler echocardiography, while significant errors can arise if measurements are estimated from standard 2D echocardiograms alone (29).

### Limitations

The results of our study need to be considered within the context of several limitations. First, all of the images were acquired by transthoracic echocardiography (TTE) rather than transesophageal echocardiography (TEE), as most of the included population were children who cannot tolerate TEE examination. Additionally, patients did not undergo cardiac computed tomography or magnetic resonance imaging, which can provide more detail information of ASD anatomy. Second, the training and testing dataset is based on images obtained from children. Despite the low prevalence, the algorithm performed very well to identify and quantify the sizes of these ASDs. This indicates that the absolute size of the heart does not influence accuracy of the model since the images are ultimately scaled to the same pixel dimensions with adequate special resolution. Third, limited by the retrospective nature, the study included a relatively small number of patients. Although our model achieved good performance in the external test set, testing of the model in a prospective multi-center cohort is warranted. Finally, the “black box” problem of our DL algorithm poses an inherent impediment to acceptance into clinical practice because of the opaqueness on how diagnoses are made. To overcome this limitation, we implemented an algorithm which provided keyframe selected by the DL model and identified the endpoints of defect and the septum on the images. This is intended to promote physician confidence in the model-based diagnoses and measurements. Even then, it is emphasized that the algorithm is intended to assistant, not replace, physician decision making.

## Conclusion

We developed and validated a novel deep learning model applicable to color Doppler echocardiography for automatic detection and quantification of atrial septal defect and rim sizes. This model has the potential to improve the accuracy and efficiency of color Doppler echocardiographic screening and quantification of ASDs.

## Perspectives

### Competency in patient care and procedural skills

Echocardiography is the most commonly used non-invasive imaging tool for detection and quantification of atrial septal defects. Manual evaluations of echocardiographic videos required highly skilled clinical experts and is a time-consuming process.

### Translational outlook

Algorithms based on deep learning approaches have the potential to automate and increase efficiency of the clinical workflow for detecting atrial septal defects and measuring the size of defect and the residual rim.

## Data Availability

The original contributions presented in the study are included in the article/**Supplementary Material**, further inquiries can be directed to the corresponding author/s.
