# Supplementary material for: Echocardiography-based AI for detection and quantification of atrial septal defect
Source: Front Cardiovasc Med. 2023 Mar 10;10:985657. doi: 10.3389/fcvm.2023.985657 (PMC10160850; doi:10.3389/fcvm.2023.985657)
Supplement: Supplementary file 1 [file Table1.docx]

**Supplemental Material**

**Echocardiography-based AI for Detection and Quantification of Atrial Septal Defect**

**Authors:**

Xixiang Lin, MD,^1,2^^#^ Feifei Yang, MD,^2#^ Yixin Chen, MS,^3#^ Xu Chen, MD,^1,2^ Wenjun Wang, MM,^2^ Wenxiu Li, MD,^4^ Qiushuang Wang, MD,^5^ Liwei Zhang, MD,^5^ Yujiao Deng, MD,^2^ Haitao Pu, MS,^3^ Xiaotian Chen, P_H_D,^3^ Xiao Wang, MD,^1,2^ Dong Luo, MD,^1,2^ Peifang Zhang, P_H_D,^3^ Xin Li, MD,^6^ Daniel Burkhoff, MD, P_H_D,^7^ Kunlun He, MD, P_H_D,^2^*

**Table of Contents**

**Table S1……………………………………………………………………………… 2**

**Figure S1…………………………………………………………………………….. 3**

**Figure S2…………………………………………………………………………….. 4**

**Figure S3…………………………………………………………………………….. 5**

**Figure S4…………………………………………………………………………….. 6**

**Supplemental figure legends……………………………………………………….. 7**

**Table S1.** Normalized confusion matrix of comparation between expert suggestion and AI prediction on treatment

| Accuracy = 85.4% | | AI prediction with Guideline | |
| --- | --- | --- | --- |
|  |  | Transcatheter | Non-transcatheter |
| Expert suggestion  with Guideline | Transcatheter | 14 | 7 |
|  | Non-transcatheter | 0 | 27 |

**Figure S1.** Normalized Confusion Matrix of View Selection Model.

**
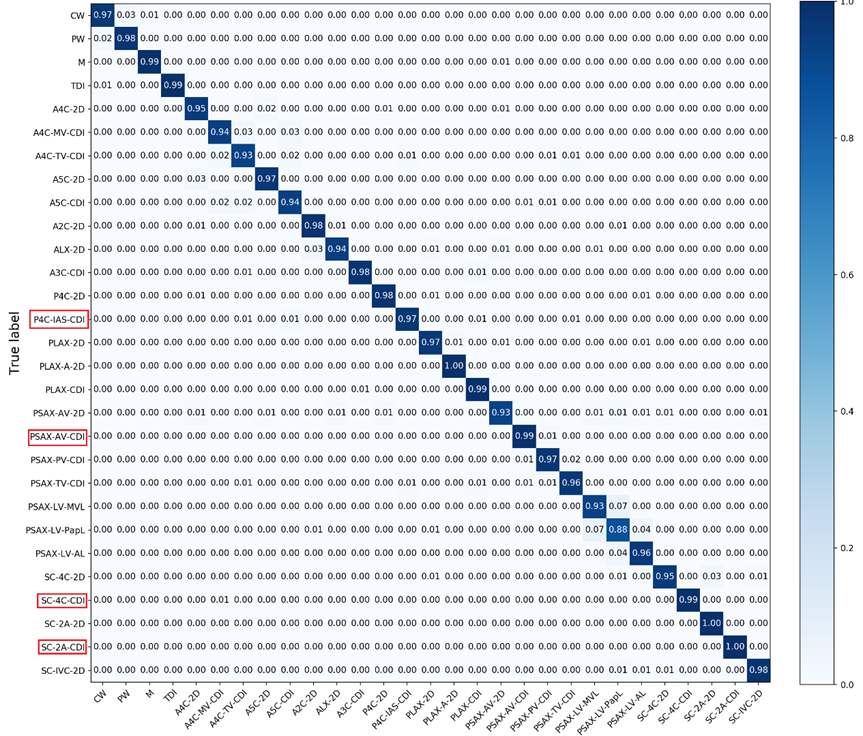
**

**Figure S2.** Examples of Key Frames Automatically Identified by the ASD Detection Model.

**
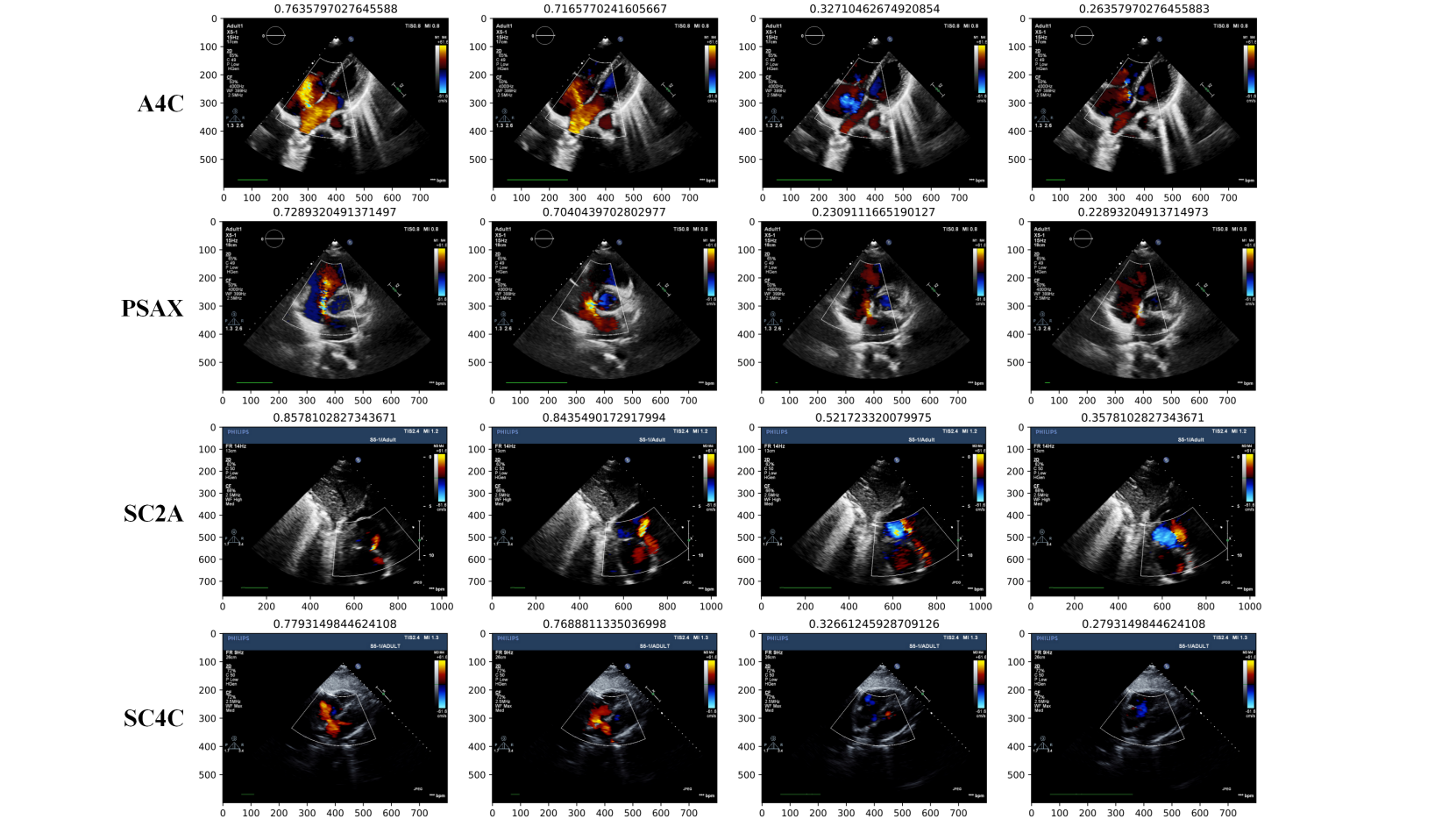
**

**Figure S3.** The Relationships between the Absolute Difference between Automated and Manual Measurements for Septum Length (upper row) and Defect Size (lower row) as a Function of the Confidence Values.

**
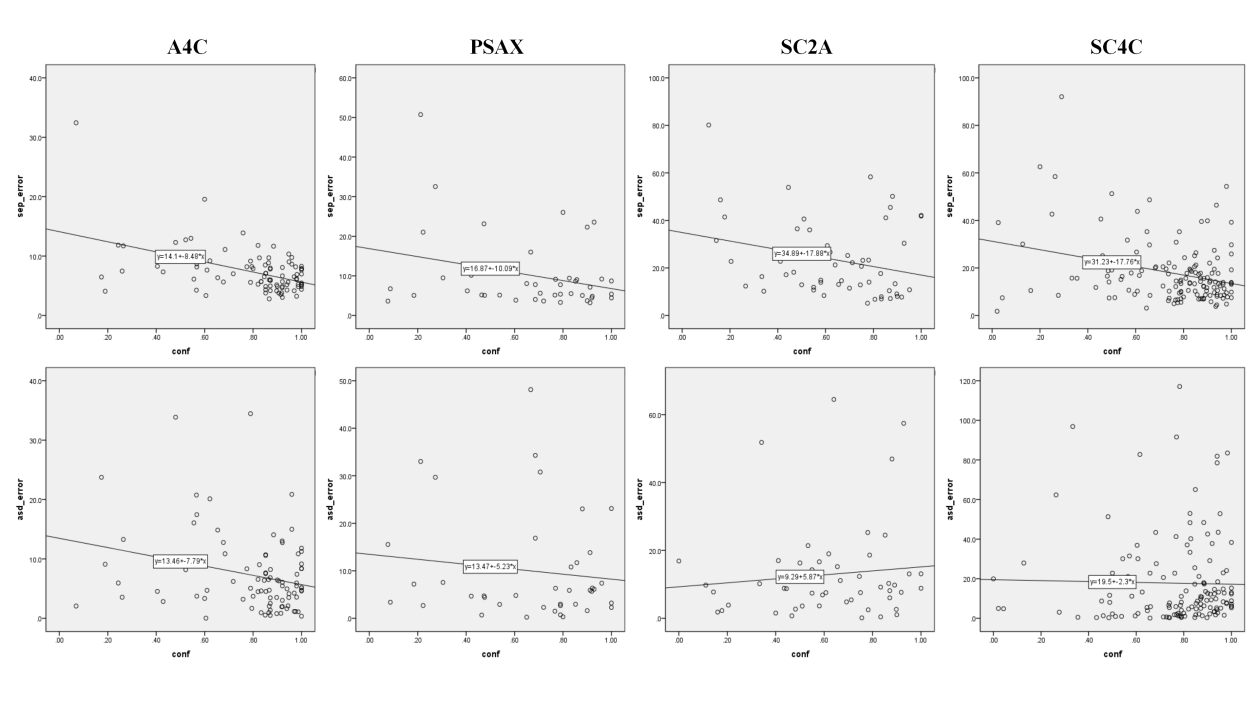
**

**Figure S4.**Comparisons of Quantitative Metrics Derived from the Experts Based on Bland and Altman Analysis

Bland-Altman plots compare the experts’ measurements for septum length and defect size in A4C, PSAX, SC2A and SC4C view. Abbreviations as in Figure 1.


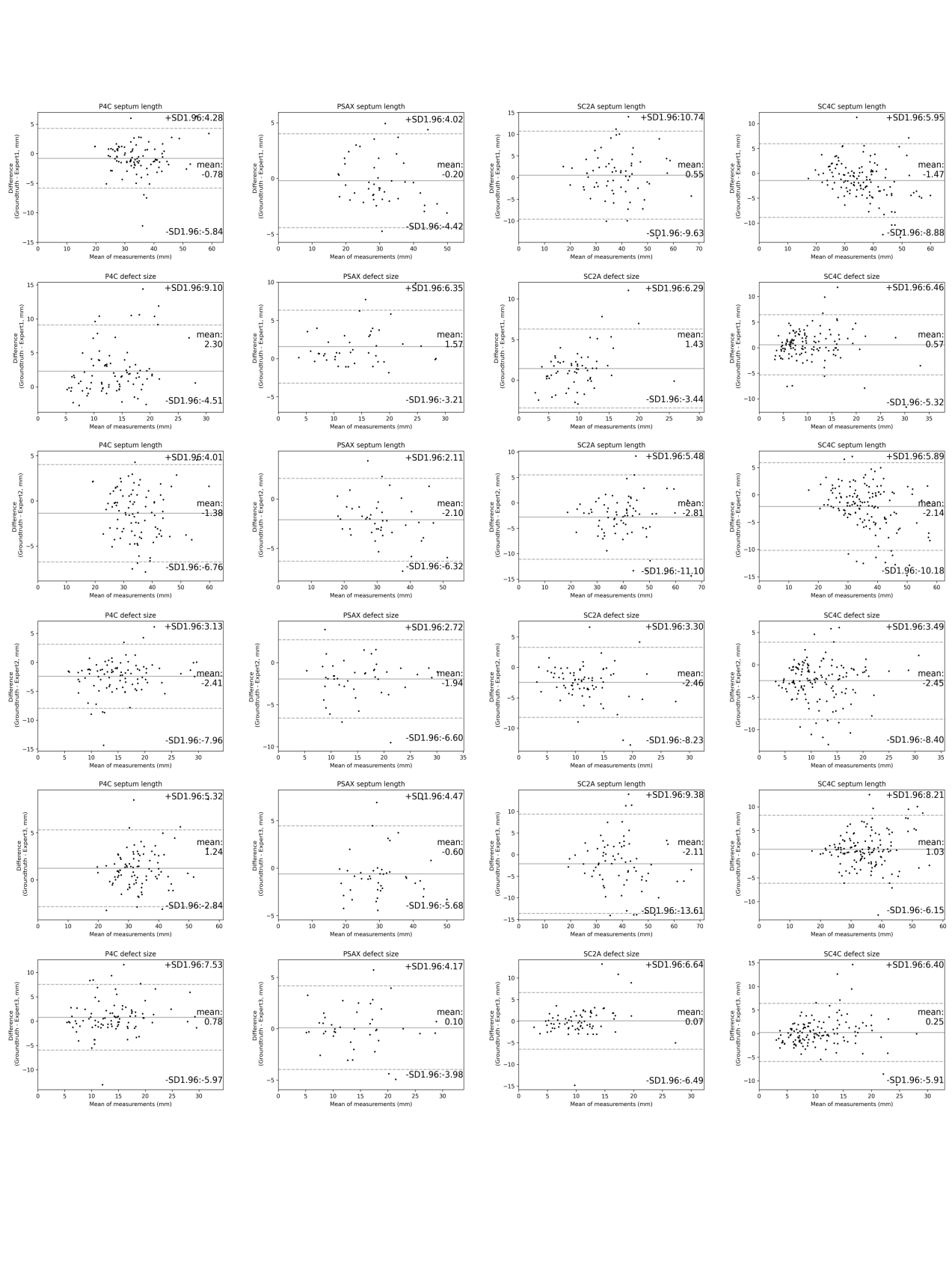


**Supplemental figure legends**

**Figure S1. Normalized Confusion Matrix of View Selection Model.**

The marked red views are required for this analysis.

**Figure S2. Examples of Key Frames Automatically Identified by the ASD Detection Model.**

The number above each image is the probability of the presence of an ASD generated by the ASD detection model. The frame with highest probability is chosen as the key frame for final diagnosis.

**Figure S3. The Relationships between the Absolute Difference between Automated and Manual Measurements for Septum Length (upper row) and Defect Size (lower row) as a Function of the Confidence Values.**

**Figure S4. Comparisons of Quantitative Metrics Derived from the Experts Based on Bland and Altman Analysis**

Bland-Altman plots compare the experts’ measurements for septum length and defect size in A4C, PSAX, SC2A and SC4C view. Abbreviations as in Figure 1.
